# Body objectified? Phenomenological perspective on patient objectification in teleconsultation

**DOI:** 10.1007/s11019-023-10148-w

**Published:** 2023-04-08

**Authors:** Māra Grīnfelde

**Affiliations:** University of Latvia Institute of Philosophy and Sociology, University of Latvia, Kalpaka boulevard 4 – 322, Riga, 1050 Latvia

**Keywords:** Phenomenology, Teleconsultation, Quality of health care, Lived body, Objectification

## Abstract

The global crisis of COVID-19 pandemic has considerably accelerated the use of teleconsultation (consultation between the patient and the doctor via video platforms). While it has some obvious benefits and drawbacks for both the patient and the doctor, it is important to consider—how teleconsultation impacts the quality of the patient-doctor relationship? I will approach this question through the lens of phenomenology of the body, focusing on the question—what happens to the patient objectification in teleconsultation? To answer this question I will adopt a phenomenological approach combining both insights drawn from the phenomenological tradition, i.e., the concepts of the lived body and the object body, and the results from the phenomenologically informed qualitative research study on the patient experience of teleconsultation. The theoretical background against which I have developed this study comprises discussions within the field of phenomenology of medicine regarding the different sources of patient objectification within clinical encounter and the arguments concerning the negative impact that objectification has on the quality of care. I will argue that a factor that has frequently been identified within phenomenology of medicine as the main source of patient objectification in clinical encounters, namely, the internalized gaze of the clinician, is diminished during teleconsultation, increasing patient’s sense of agency, decreasing her sense of alienation and opening up the possibility for a closer relationship between the patient and the health care provider, all of which lead to the transformation of the hierarchical patient-health care professional relationship.

## Introduction

The global COVID-19 pandemic has considerably accelerated the use of online communication forms, including the use of teleconsultation, or consultation between the patient and the doctor via video platforms. During recent years, health care systems globally have been resorting to telemedicine^1^ to provide continuous medical care to patients in their homes, thus avoiding COVID-19 exposure risks (Bashshur et al. 2020; Hollander et al. 2020). Teleconsultations have been offered by a variety of health care specialists to their patients for chronic disease reviews, counseling or other talk therapy, administrative appointments (for example, for sick notes), medication reviews, and triage when a telephone call is deemed to be insufficient (Greenhalgh et al. 2020). Responding to this situation, a number of research studies have emerged that discuss the benefits and challenges of teleconsultation on the basis of a variety of methodological approaches (Connolly et al. 2020; Feijt et al. 2020; Turner et al. 2022). It has been established that as a general rule, teleconsultation provides increased convenience and greater flexibility and offers increased accessibility to health care; however, it also raises challenges, some of which has to do with a lack of patient technical skills or of the technology itself, while some of which has to do with confidentiality problems (Holtz 2021; Feijt et al. 2020, Turner et al. 2022).

While these studies provide valuable insights into the benefits and challenges of teleconsultation, little has been written about the possible impact that teleconsultation has on the quality of the patient‒physician relationship (see Pols 2012, Heckemann et al. 2016, Gomez et al. 2021, García et al. 2022, Frittgen and Haltaufderheide 2022, Bizzari 2022). Moreover, no qualitative research has yet considered the impact of teleconsultation on the objectification of the patient, which is a praxis referring to the health care provider’s focus on the patient as a mere object of medical manipulation and is claimed by medical sociologists, philosophers of medicine and phenomenologists of medicine to have a negative impact on the quality of care (Svenaeus 2021; Timmermans and Almeling 2009; Marcum 2004). Members of the latter group, namely, phenomenologists of medicine,^2^ have devoted a great deal of attention to this issue in recent decades and have used insights drawn from phenomenological philosophy to illuminate both the concept of objectification and the sources of patient objectification within the clinical encounter, with the general consensus being that aside from medical technology itself, the clinical gaze is the main source of the patient’s objectification, leading to the sense of alienation on the part of the patient.

Despite these extensive discussions within the phenomenology of medicine, the question of what changes occur in patient objectification in the context of *online* clinical encounters has not yet been addressed.^3^ The aim of my paper is to argue that contrary to the assumption made within the contemporary phenomenology of medicine that the clinical encounter is a source of patient objectification, in teleconsultation, the objectification of the patient is significantly reduced. More concretely, I will argue that a factor that has frequently been identified as the main source of patient objectification in clinical encounters, namely, the internalized gaze of the clinician, is significantly weakened during teleconsultation, thus decreasing the patient’s sense of alienation in that context. I will show that the reason for this effect lies in both the shift in the patient’s perspective on the doctor (teleconsultation offers the possibility for the patient to experience the doctor as a human being and thus to form a closer relationship with her) and the shift in the doctor’s perspective of the patient (teleconsultation offers the possibility for the doctor to focus on the patient’s illness story instead of her physical body). Furthermore, I will show that the diminished sense of patient objectification that occurs in the context of teleconsultation has a significant impact on the dynamics of the clinical encounter—the traditional hierarchical clinical encounter is transformed into a more horizontal encounter, in which context the patient no longer plays the role of a passive recipient and instead becomes an active participant in her own healing process, whereas the doctor is no longer viewed only as a person in a position of power but also as an approachable human being, all of which contribute to more patient-centered care.

I will approach this issue from a phenomenological perspective by combining insights drawn from the phenomenological tradition, i.e., the concepts of the lived body and object body, with the results of my phenomenologically informed qualitative research study on patient experiences with teleconsultation. The conceptual lens offered by phenomenology, namely, the concepts of the lived body and the object body, has been used by a number of phenomenologically oriented researchers to illuminate a variety of lived experiences of patients, such as the experience of hemispatial neglect after stroke (Klinke et al. 2014; Klinke, et al. 2015), depression (Fuchs 2013), anorexia (Fuchs 2022), breast cancer (Slatman 2016), facial limb absence (Yaron et al. 2017), and many others. Recently, the phenomenological lens has also been used to investigate patient experiences with online psychotherapy. More concretely, García et al. (2022) and Bizzari (2022) used the phenomenological concepts of embodiment, intercorporeality and interaffectivity to ground qualitative research on patient (and doctor) experiences of online psychotherapy to explore how the embodied processes that take place within clinical encounters are modified in the context of online psychotherapy. While neither García et al. nor Bizzari focused on the question of patient objectification in online psychotherapy, some of the results of their studies support the insights provided in this paper, thus strengthening the validity of this research. For example, García et al. (2022) argued that online psychotherapy increases patients’ control and responsibility (p. 15), transforms the asymmetrical patient-clinician relationship into a more symmetrical interaction (p. 12) and tends to increase the amount of verbal communication that occurs between patients and doctors in online meetings (p. 13). Bizzari (2022) also emphasized the importance of verbal communication between the doctor and the patient (p. 56). Both research studies argued that the ability of the patient to observe her own image on the screen in an online context has a negative impact on the clinical encounter (Bizzari 2022, p. 4; García et al. 2022, p. 14). As I will show, with regard to the discussion of the patient objectification, this finding illustrates the loss of the patient’s lived body and her transformation into the object body. It must be noted, however, that both García et al. (2022) and Bizzari (2022) focused exclusively on online psychotherapy, while the results of the research presented in this paper consider both mental and physical health care in an online context. This difference accounts for some of the discrepancies between their results and those of this study.^4^

It is also important to note that both García et al. (2022) and Bizzari (2022) are skeptical regarding the possibilities of online psychotherapy, highlighting the disturbances that occur in clinical relationships due to the videoconferencing medium. Without denying the fact that negative consequences for the clinical relationship can occur when that relationship is moved online, focusing on patient experiences with teleconsultation through the lens of patient objectification presented in this paper both allows us to see some previous findings in a more positive light—for example, by showing that the emphasis on verbal communication in teleconsultation is not necessarily merely negative (Bizzari 2022)—and to generate new findings regarding the nature of online clinical encounters—for example, by showing that teleconsultation leads to a diminished sense of patient alienation and a closer relationship between the patient and the health care provider. In addition, the results presented in this paper can both improve our understanding of some previous findings regarding teleconsultations^5^ and challenge some previous ideas regarding online clinical encounters, for example, the claim made by Bizzari (2022) that the lack of physical bodies in online clinical encounters leads to a disruption of the clinical relationship and the claim made by phenomenologists of medicine (Toombs 1992; Carel 2016) that the clinical encounter in general is alienating for the patient.

I will start by referring briefly to the phenomenological distinction between the lived body and the object body and by classifying the possible sources of objectification within the clinical encounter that have been proposed by phenomenologists of medicine. Thereafter, with the help of the results of my phenomenologically grounded qualitative research study of patient experiences with teleconsultation, I will argue that such objectification is significantly reduced in the context of teleconsultation.

## Phenomenology of the body and sources of patient objectification

It has been pointed out by phenomenologists of medicine that a major factor influencing the quality of the patient‒physician relationship is the focus of a health care provider on the patient as a mere physical body that needs to be fixed (Toombs 1992, p. 87; Carel 2016, p. 221; Svenaeus 2021), which leads to the objectification of the patient as a disease entity and imparts feelings of alienation and loss of agency on the part of the patient.^6^ As Havi Carel writes, “Health professionals often view the body as thematized and objectified, focusing on a particular organ or function in order to understand it as a medical object. But for the patient, the awareness of her body as an object is secondary to her subjective experience of receiving healthcare” (2016, p. 220). The patient is not only a disease entity that needs to be fixed but also a person who experiences the disease (an ill person). Here, two perspectives on the body are at a play, an external one (the body as an object, accessible not only to oneself but also to others) and an internal one (the body as a subject, directly accessible only to oneself). Both perspectives are included in the concept of the body found in phenomenological philosophy. According to the phenomenological approach, “the body is not merely an object of experience that we see, touch, smell, etc. Rather, the body is also a principle of experience; it is that which permits us to see, touch, and smell, etc.” (Gallagher and Zahavi 2008, p. 135).

This duality in the experience of the body is illustrated by the well-known distinction made by Husserl (2000) between *Leib* (lived body) and *Körper* (object body).^7^ Husserl illustrated this duality by reference to the example of touching one’s left hand with one’s right hand (2000, p. 152). We experience this hand in a dual way—the left hand is felt not only as an object with a certain extension and location in the world, among other things, but also as a bearer of sensations (Husserl 2000, p. 152–153, 159–160, 168–169) and as the seat of free movement, which is characterized by the faculty of “I can” (p. 159). As a bearer of sensations, the body is characterized by its inseparability from the self (p. 157). When I feel hot or cold, pain or pleasure, I feel myself feeling. Thus, the lived body expresses the experiential unity between the self and the body. On a theoretical level, this experiential unity between the body and the self highlights a rejection of the Cartesian dualism of the body and the mind, while on the experiential level, it highlights the fact that in ordinary circumstances, there is no distance between myself and my body. Merleau-Ponty describes this in the following way: “(…) I am not in front of my body, I am in my body, or rather I am my body” (2002, p. 151).

As the seat of free movement, the lived body refers to the embodied agency of the subjectivity in its involvement in the world. While this aspect of embodiment was introduced by Husserl, it was the main focus in Merleau-Ponty’s phenomenology of the body. Merleau-Ponty developed Husserl’s insights into the body as an embodied consciousness of “I can” further, arguing that the lived body, as the seat of embodied agency, is responsible for the appearance of the world (2014, p. 139, 147). One endows one’s world with meaning through bodily perception and movement (seeing, hearing, changing one’s location, grasping, etc.). It is through our body that we live and that we interact with, and experience ourselves, the world and other people. Merleau-Ponty describes this aspect of embodiment by reference to a motor or original intentionality: “Consciousness is originarily not an ‘I think that’, but rather an ‘I can’” (2012, p. 84). In ordinary circumstances, we do not experience our bodies as objects. We are instead directed toward projects in the world through our bodies. The lived body is thus an ecstatic body, i.e., that from which we perceive and act, itself remaining in the background (Leder 1990, p. 58).

In contrast, the experience of one’s body as an object presupposes the conscious awareness of one’s body and leads to the experience of a distance between the body and the self, which can evoke feelings of alienation.^8^ This focus on one’s body as an object can be either welcome (as, for example, in physical exercise, sexual arousal, dance, wanted pregnancies, etc.) or unwelcome (as, for example, in unwanted physiological reactions, physical damages or feelings of shame) (Toombs 1992, p. 62; Zeiler 2010, p. 338–340; Leder 1990, p. 84–85). The source of unwelcome objectification can be either the body itself (for example, pain or illness) or the other (for example, a judgmental health care professional). Regarding the latter, Jean-Paul Sartre in his philosophy (2001) emphasized that we can experience our bodies in the mode of being for another (*pour autrui*) or as perceived by others. To give an example, Sartre writes, “We often say that the shy man is ‘embarrassed by his own body.’ Actually, this expression is incorrect; I cannot be embarrassed by my own body as I exist it. It is my body as it is for the Other which may embarrass me” (2001, p. 353). For this reason, the other plays an important role in the experience of oneself.

According to phenomenologists of medicine, an unwelcome objectification of the patient’s body frequently occurs within the clinical encounter. In addition to already experiencing bodily objectification due to illness, objectification is furthered both because the patient perceives her body (or its part) through the internalized gaze of the other (health care professional) and because the patient encounters her body through medical technology. Regarding the first, a health care professional’s focus on a patient’s body as a mere biological organism (object body) can intensify the patient’s own experience of her body as an object (which is usually already present to some extent due to the unwanted bodily reactions and feelings brought about by illness) and increase the accompanying feelings of alienation. Regarding the second, an encounter with medical technology can also lead to the perception of oneself as a mere object of investigation rather than a living, suffering body (Toombs 1992, p. 94). Carel writes: “Seeing one’s tumour as a set of CT images or aligning your limbs for a bone density scan can make the objecthood of the body prominent in one’s experience. These objectifying experiences may lead to a sense of alienation from one’s body and to treating that body as an aberrant object over which one has little control (2016, p. 221).^9^ In this quote, aside from a sense of alienation, Carel mentions another important consequence of one’s transformation to objecthood, namely, the loss of control. When a patient experiences herself as a mere object body, she no longer feels able to effectively control what happens to her (Toombs 1992, p. 95). This in turn leads to passivity on the part of the patient, a loss of autonomy and a loss of personal responsibility over her own healing process (Leder 1984, p. 36; Toombs 1992, p. 85).

Considering the aforementioned two sources of objectification in clinical encounter, i.e., the medical gaze and medical technology, in this paper I will focus on the patient’s experience of objectification as a result of the gaze of the clinician and technology itself, arguing that the factor that has been identified most frequently as the main source of patient objectification within clinical encounters, namely, the internalized gaze of the health care provider, is weakened in the context of teleconsultation. While I approach this issue from the perspective of the patient experience, the results of the study can be useful for both patients and health care professionals. I will show that the main source of patient objectification within clinical encounters, namely, the internalized gaze of the health care provider, is weakened in the context of teleconsultation, thereby offering new possibilities for clinical interaction, such as an increased sense of control and personal responsibility on the part of the patient as well as a closer connection to the health care professional. Knowledge of these new possibilities of interaction can be employed by health care professionals to improve both the healing process both online and in person.

## Methodology

The research study involved 14 semi-structured interviews with people who had had at least one online video consultation with a medical specialist within one year prior to the interview.^10^ These specialists included specialists in both mental and physical health.^11^ Of the participants, 11 were women and 3 were men, and they ranged in age from 24 to 39 years old. Participants were recruited using several patient organization platforms in Latvia as well as by applying a snowballing approach within the social network of the researcher. Informed consent was discussed with and obtained from each participant at the beginning of each interview, and all data used in this paper and elsewhere are anonymized. The research study received approval from the University of Latvia Human and Social Sciences Ethics Committee. Due to safety restrictions during COVID-19, all interviews took place over the videoconferencing platform Zoom^12^ and lasted between 50 and 90 min each. They were recorded and then transcribed verbatim. After the transcription of the interviews, an analysis began using NVivo 12 plus program to facilitate codification.

**Table 1:** Overview of the participants

Number, name	Sex, age	Specialist	Previous in-person familiarity with the specialist	Length of a single consultation	Number of consultations	Videoconferencing platform used
1. Alice	F/37	Internist	No	20 min	1	doxy.me
2. Vilma	F/24	Psychotherapist	No	1 h	6	Skype
3. Andrea	F/39	Otolaryngologist	No	30 min	1	Zoom
4. Thomas	M/31	Neurologist	No	50 min	1	Zoom
5. Dana	F/35	Psychotherapist	Yes	1 h	3	WhatsApp, Zoom
6. Julie	F/28	Gastroenterologist	Yes	20 min	1	Zoom
7. Mark	M/24	Physiotherapist	No	40 min	1	Zoom, MS Teams
8. Sophia	F/26	Internist	No	30 min	1	Skype
9. John	M/35	Psychotherapist	Yes	45 min	10	Zoom
10. Louisa	F/35	Psychotherapist	No	1 h	10	Zoom
11. Agnes	F/33	Psychotherapist	Yes	45 min	6	Skype, WhatsApp
12. Anna	F/37	Family doctor	No	10 min	1	Babylon
13. Maria	F/32	Psychotherapist	Yes	1 h	9	WhatsApp
14. Christina	F/33	Midwife	No	1 h–1.5 h	5	Zoom, WhatsApp

In the study, I integrate phenomenological philosophy with qualitative research. The main concern of phenomenology since its beginnings (in the early twentieth century) has been a focus on lived experience prior to any scientific explanations, which was already expressed by Husserl (2001 [1900/1901]) in his famous appeal to go back to the “things themselves” (p. 168). According to Dan Zahavi, this dictum “should be interpreted as a criticism of scientism, and as a call for a disclosure of a more original relation to the world than the one manifested in scientific rationality” (2008, p. 664). It is a call for a return to the world of experience as it appears to the experiencing subject from a first-person perspective. However, it is important to emphasize that phenomenologists do not simply provide everyday descriptions of experience given from a first-person perspective. Rather, they attempt to identify the structure or essence of the lived experience in question. As Gallagher and Zahavi maintain, “Phenomenology has as its goal, not a description of idiosyncratic experience—‘here and now, this is just what I experience’—rather, it attempts to capture the invariant structures of experience” (2008, p. 28). By focusing on the structure of experience, phenomenology claims to offer a transcendental account of the possibility of the experience in question. This does not mean that it ignores the facticity of this experience. Although phenomenology is a study of essences, it is also “a philosophy that places essences back within existence and thinks that the only way to understand man and the world is by beginning from their “facticity”” (Merleau-Ponty 2002, p. [lxx]). This means that the essence of any experience is accessible only through the concrete socioenvironmental context of its occurrence.

It is precisely because of this emphasis on the lived experience of the person herself that phenomenological philosophy can and has been integrated with qualitative research. In the context of the application of phenomenology to health care, this combination has proven to be especially fruitful. Researchers have been focusing on the lived experiences of a variety of illnesses (for example, the experience of depression, cerebral palsy, schizophrenia, anorexia nervosa), child birth, organ transplantation, and the experience of shame and other emotions within the clinical encounter. In designing this research study, conducting interviews and analyzing the data, I used the Phenomenologically Grounded Qualitative Research (PGQR) methodology (Køster and Fernandez 2021) and the “Phenomenological Interview” (PI) framework (Høffding and Martiny 2016).^13^ Both of these approaches diverge significantly from other well-known methodologies that combine qualitative research with phenomenology (for example, those of Giorgi (2009), Smith et al. (2009), and van Manen (2016)) because they draw on philosophical phenomenology’s concepts rather than on its methods. Køster and Fernandez (2021) described this use of phenomenology’s concepts as a phenomenological grounding of qualitative research, arguing that this grounding allows researchers to focus on the specific modifications of certain structural dimensions of human existence. Recently, various research studies have used one or more of the core phenomenological concepts, for example, embodiment, intercorporeality, body schema, body image, selfhood, intentionality, affectivity, spatiality and temporality, to ground qualitative research mostly, although not exclusively, in the fields of psychopathology and health care (see: Klinke et al. 2014; Klinke et al. 2015; Slatman 2016; Yaron et al. 2017; Ekdahl and Ravn 2022; García et al. 2022).

In this study (in terms of designing the research study, conducting interviews and analyzing the data), I drew on the phenomenological distinction between the lived body and the object body to illuminate how dimensions of the human existence that are expressed in these concepts are affected in teleconsultation. The interview process itself was largely inspired by an account presented by Høffding and Martiny, who maintain that “in the interview process one should be aware of one’s phenomenological commitments, take up an empathetic, reciprocal and second-person perspective when encountering the subject, and ask specific open questions in order to get descriptions that are as detailed as possible” (2016, p. 558).^14^ The study’s interview guide included a few predefined focus points structured around the categories associated with the concept of embodiment. The process of analysis included three steps. The first step was to bracket from the transcriptions all nonessential material, such as instances where participants completely strayed from the topic. The second step was to structure the descriptions of patient experiences with teleconsultation into several categories. The third step was to further analyze the descriptions contained in some of the key categories (such as categories of the “the lived body” and “the object body” in the context of this study) by situating them within theoretical discussions in the field of the phenomenology of medicine about the distinction between illness and disease, the objectification of the patient and the possible impact that this objectification has on the nature and quality of the patient‒physician relationship.

The analysis of the data was conducted in accordance with the criteria of internal and external phenomenological consistency (Høffding and Martiny 2016). Internal phenomenological consistency refers to the “ability to make comprehensible all the descriptions found in the interview. The more descriptions that can be made comprehensible under a certain phenomenological interpretation, the deeper the internal phenomenological consistency” (p. 549). External phenomenological consistency “refers to the ability of the overall account produced to work with and against already established theories of the phenomena in question” (p. 549). According to the authors of PI, external phenomenological consistency is related to the methodological step known as “intersubjective validation” (Varela and Shear 1999, p. 10), which means that “the account should be consistent with the relevant theories, but can also be in a position to challenge them” (Høffding and Martiny 2016, p. 550). In the context of this study, the level of internal phenomenological consistency was high because most of the patients’ descriptions of their experience of teleconsultation made sense when they were situated in the conceptual framework of the lived body and the object body. The level of external phenomenological consistency was also high because the results of the study both supported certain insights already expressed in previous research on patient experiences with teleconsultation (for example, the transformation of the patient-health care professional relationship into a more symmetrical relationship and the dominance of the patient’s verbal account of her problem) and challenged the dominant assumption made within the phenomenology of medicine that the clinical encounter is a source of patient objectification. Furthermore, to ensure the intersubjective validity of the research findings, drafts of the research process were presented at various medical humanities and phenomenological conferences and seminars, leading to several reinterpretations of the arguments mentioned above.

## Phenomenology of illness and disease

Phenomenologists of medicine have referred to the distinction between the lived body and the object body that is found in the phenomenological tradition to illustrate the different perspectives held by the patient and the health care professional regarding the patient’s problem and expressed in the conceptual distinction between illness and disease (Toombs 1987, p. 221; Svenaeus 2011, p. 337–338; Carel 2016, p. 15–17). Toombs has pointed out that illness and the body mean something significantly different to the patient than they mean to the physician. She writes, “This difference in perspectives is not simply a matter of different levels of knowledge but, rather, it is a reflection of the fundamental and decisive distinction between the lived experience of illness and the naturalistic account of such experience” (1992, p. 89). While the former constitutes an internal perspective on how the patient experiences her illness, the latter presupposes an external perspective on the patient’s body as a physical object and corresponds to a purely scientific anatomical/pathological model of disease (Marcum 2004; Toombs 1992; Leder 1990).

The difference in focus on the patient, either in terms of illness or disease, impacts our understanding of the goal of a clinical encounter. If the primary focus within clinical encounters is on the disease state (object body) of the patient, then the goal of a clinical encounter is primarily to make a diagnosis and to cure. If, however, the focus is on the lived experience of the illness (lived body), then the goal of a clinical encounter is healing, which denotes the restoration of the patient's integrity as a human being (including but not limited to the restoration of bodily integrity) (Toombs 1992, p. 112). If a health care provider focuses solely on the curing of ailments, then she disregards the patient’s experience of the lived body disruption (the meaning of the illness) and the suffering associated with that. This has significant implications for individuals facing chronic or incurable illness. Toombs writes: “Even in the absence of cure, and indeed even in the face of death, it is possible to promote and receive healing. The need for healing is grounded in the recognition that illness is an assault on the whole person—physical, psychological, social, and spiritual” (2019, p. 218).

For the clinical encounter to be successful (i.e., for healing to occur), the patient and the physician need to communicate with one another on the basis of a shared understanding regarding the patient's illness (Toombs 1992, p. 89). This does not mean that a health care professional should disregard the physical disease process; rather, it simply means that the physician needs to view the patient not only as a physical body (focus on the disease) but also as a lived body (focus on the illness). While there are different ways in which the physician can gain insights into the patient’s lived experience of illness (and thereby establish a shared understanding of the meaning of illness with the patient), one of them is by attending to the clinical narrative, or the story of the illness, as told by the patient herself (in contrast to focusing solely on her medical history) (Toombs 1992, p. 103). Toombs writes, “If one is to understand the lived experience of illness, comprehend what the disorder means to the patient (and thus address directly the patient's disorder and suffering), then it is clear that one has to go beyond objective, quantifiable, clinical data and elicit the patient's illness story” (Toombs 1992, p. 105). In addition to facilitating the healing process, the focus of health care professionals on the lived experience of the patient’s illness mitigates some of the dehumanizing aspects of medical care, such as the treatment of the patient as a mere object body or disease entity and subsequent passivity on the part of the patient (Leder 1984, p. 36; Toombs 1992, p. 86; Marcum 2004, p. 314).

## Patient objectification during teleconsultation

What happens to patient objectification in a teleconsultation? Does the teleconsultation contribute to the objectification of the patient, leading to a dehumanizing attitude toward her? Or does the teleconsultation somehow offer the possibility for the patient to experience herself as the lived body, thus contributing to an empathetic relationship between the patient and the doctor? What is the role of videoconferencing technology in the patient experience of herself? To answer these questions, I will refer to the results of my qualitative research study of the patient experience of teleconsultation. In doing so, I will focus on three themes, which are closely intertwined, namely, the patient’s experience of herself in the teleconsultation, her perceived attitude of the health care provider and the impact that the videoconferencing platform itself has on the patient’s objectification.

### Experience of the self as a lived body

How does the patient experience herself during teleconsultation? While the experience of the participants in this research study was not uniform (as will be illustrated later), most of them said that they did not pay attention to themselves or their bodies during teleconsultation. One participant (Andrea) describes it in this way: “[during teleconsultation] I don’t have to focus on my body so much. Instead, I can focus on the problem, which I have.” Another participant (Alice) said: “I can focus only on what I am saying and on what the doctor is saying.” Or, another one (Anna): “I concentrated on the conversation we were having, because my problem was the most important thing for me [during the consultation], I wanted to talk about it and resolve it.” This focus on the discussed problem indicates that the patient does not experience her body as an object during teleconsultation; instead, the patient experiences herself as a lived body, through which she is directed toward the projects in the world—in this case, toward the discussed problem.

The argument that patients experience themselves as lived bodies and not as object bodies during teleconsultation is supported by the fact that their sense of agency is increased during teleconsultation. This sense of agency refers to the experiential possibilities of the patient’s lived body (both affective and social), which are expressed in the “I can.” For example, interview material shows that the patient’s sense of control increases during teleconsultation.^15^ One participant (Vilma) describes it in the following way:

The fact that I am behind the screen allowed me to feel safe, at least in the sense that at any time I have a power over what will be said, at any time I can mute the doctor, I can take out my earplugs, I can turn away, I can turn off [my computer], if I don’t like something. In addition, this gives me a sense of control over the situation.

Andrea expressed her experience: “What else? Oh, the ability to have control because I know that if I don’t like something, I can turn off everything. I cannot turn off a live human being, but I can turn off the computer.” The sense of control applies not only to the control over the situation in general but also over the environment. Thomas says:

I prefer videoconferencing to real-life consultations, with or without COVID, because [in videoconferencing] I can be in a controlled environment and I can prepare more.

This control over the environment includes not only the possibility of choosing the patient location for the consultation and deciding what can be seen by the health care professional but also the possibility of ensuring the availability of necessary items (such as pens and paper, water and tissues). In addition, patients mentioned some new potential behaviors that occur during teleconsultation (in comparison to real-life consultations), such as the ability to hide things from the doctor (for example, nervous hand gestures or using one’s phone) and the ability to multitask (for example, finding information on the internet while talking to the doctor).

The patient’s sense of agency is also expressed in her sense of responsibility, which is increased in teleconsultation. Some participants referred to the increased responsibility of establishing a secure and private environment (including responsibility over technical issues), and others mentioned feeling increased responsibility over their own well-being. Regarding the latter, some patients mentioned assuming greater responsibility over taking care of themselves in cases of emotional distress (they need to find ways to calm themselves down without the sometimes helpful physical presence of the other), while others mention feeling an increased sense of responsibility to communicate their health problem as best as possible. Overall, as one participant (Dana) said:

I had a joke with my friends that we should pay at least 10 euros less for a teleconsultation because we have to assume the responsibility over it as well. Well, responsibility for things that the doctor would have to be responsible for [in real life consultation]. There [in real-life consultation] we would just need to show up and nothing more.

The last sentence expresses the perceived need of the patient to be more actively involved in the teleconsultation in comparison to the more passive role she assumes in traditional real-life consultations.

### Objectification through the gaze of the health care professional

The interview material shows that the two general sources of the objectification mentioned in the introduction, namely, the body itself with its physiological reactions and the gaze of the other, are weakened in teleconsultation. Regarding the former source of the objectification (the body itself), one participant (Maria) described it in the following way:

[During teleconsultation] I am usually sitting on a floor and there have been times when I am so absorbed in the conversation (and I am sitting in a lotus position)^16^ that after the end of the consultation I am planning to get up and go somewhere, but I only then realize that my legs are numb.

Another participant (Vilma) said, “I had to change my shirt [after the consultation] because it was damp with perspiration. I did not realize that during the consultation, only afterward.” The focus on the discussed problem during teleconsultation overshadows some of the physiological reactions of the body to the point that the patient is not even aware of them.

The latter source of the objectification (the internalized gaze of the health care professional) is also weakened in teleconsultation. As one participant (Andrea) says:

[in an on-site consultation] I cannot focus fully on the consultation and realize my own interests, because I am occupied with thoughts about how I look, if everything is okey, if I act normally, if I don’t sit in a wrong chair. However, now [in teleconsultation] all of these concerns disappear, now there is only the screen and I can feel completely free and I can explain my problem.

This suggests that the objectifying attitude of the health care professional is diminished in a teleconsultation. What could be the reason for this? Based on the interview material, I suggest that the reason for this is found in the very nature of the online environment, which involves the absence of the physical body of the other. Patients focus less on their own bodies because they feel less affected by the “objectifying gaze” of the health care professional. They feel less affected by this gaze because they are aware of the fact that it is very difficult (or even impossible) for health care professionals to focus on patients’ bodies during a teleconsultation.

There is, however, another reason for the lack of objectification of the patient, and this is the perceived image of the health care professional during teleconsultation. Even if the health care professional cannot focus on the patient’s actual physical body or inspect the virtual expressive body, she might still be able to objectify the patient, for example, by focusing only on the medical records available to her (if there are any) or by having a judgmental attitude. Without excluding the possibility of such occurrences, participants in the study did not report any of these occurrences. In addition to perceiving the health care professional as literally unable to focus on patients’ physical bodies, some patients perceive her as more empathetic, approachable and interested in their problems. This can be explained by two things. First, it can be explained by the actual shift in health care professionals’ attitudes toward their patients that is initiated by the online environment and, second, it can be explained by certain features of the online environment.

Regarding the former, the lack of access to the patient’s physical body in online clinical encounters facilitates a shift in the health care professional’s perspective, i.e., in the absence of the patient’s physical body and due to limited access to the patient’s expressive body in the teleconsultation (only the head of the patient is usually visible), the health care professional has no other option than to focus on the illness story of the patient. In contrast to the in-person clinical consultations, where health care professionals often focus exclusively on the patient’s diseased body or its part, which evokes the experience of objectness and the corresponding feelings of alienation on the part of the patient, during a teleconsultation, health care professionals are forced to focus on the lived body of the patient, thus cultivating empathetic attitudes.^17^ Participants of the study expressed this by emphasizing that the experience of the health care professional is one of listening to them. One patient (Andrea) said, “I liked that she [the doctor] was forced to listen to my interpretation of my problem and did not just focus on my body from a third person perspective.” This diminished importance of the gaze of the health care professional during teleconsultation allows patients to forget about their bodies and live them.

Regarding the later, some patients perceive health care professionals as being more approachable during teleconsultation due to a particular feature of the online environment, namely, the fact that the online environment lacks significant aspects of the medical environment, such as the specific smell, medical equipment, doctor’s white coats, other personnel moving about, etc. Some participants of the research study describe perceiving the health care professional as “a human being,” rather than as a person in a position of a power. One participant (Alice) said, “You are talking to the doctor human being, and not to the doctor in a white coat in a medical establishment. I even think that this conversation is more humane.” The online environment, lacking the attributes usually associated with a medical environment, allows some patients to feel less intimidated by the entirety of the setting, including the health care professional, and to be more open about their problems, leading to an increased sense of agency. In some cases, the online clinical environment not only lacked the attributes of a habitual medical environment but it also gained new attributes. This happened when health care professionals offered consultations from their homes. This home environment of the doctor, which was sometimes visible to the patients, contributes to the perception of the doctor as being more approachable. As one participant (Julie) says:

We had a very free and open communication. I saw that the doctor was in a similar setup as me – in a bed and with a pillow behind her head. [...] The doctor’s stiff coat was missing, and I had a feeling like when I am talking to a colleague, for example. For this reason, it was much easier to talk to her [the doctor].

### Objectification through medical technology

What exactly happens in the objectification of one’s body through medical technology, which, as pointed out in the introduction, can also occur in clinical encounters? While none of the study participants encountered their own body through medical technology during teleconsultation (for example, seeing results of an X-ray on the screen), this remains a possible source of objectification, which is similar to that in face-to-face clinical encounters. There is, however, the technology of videoconferencing itself, with its particular characteristics, some of which, such as the ability to see one’s own screen image when one is talking to someone else (as in Zoom, for example) can facilitate the objectification of the patient. This possibility of seeing and observing one’s own screen image, which is absent in real-life clinical consultations,^18^ can lead to self-objectification during teleconsultation. One participant (Andrea) described this situation in the following way:

When I go to the doctor, I don’t see myself. I see myself only in the morning when I look in the mirror and make myself ready for the day. In addition, that’s it, I no longer see myself. [...] I don’t have to encounter myself anymore. However, here [in teleconsultation] I see myself constantly, and this is very weird.

This possibility of seeing oneself can lead to self-objectification. As one participant (Louisa) says, “I see myself [on the screen] and it takes my focus away […] I instantly start to think, “Oh, how I look!,” I almost feel sorry for myself. I see that I have been crying.” This feature of certain videoconferencing platforms, namely, the ability to observe oneself on the screen, can shift focus away from the discussed problem to the body itself. In other words, it can disrupt the lived body of the patient, leading to the experience of oneself as an observable object.

In addition, self-observation on the screen intensifies the real or imagined gaze of the other. As one participant (Thomas) says:

A big problem [during teleconsultation] is the fact that I see myself. I don’t want to be able to see myself. In addition, what bothers me is that I am aware that others see me as well. [...] It bothers me that others can see how I look and how I act. This makes me focus on the screen to see how I look and all that. And this causes anxiety.

It should be noted here that the health care professional does not have to actually look at the patient in order for the patient to experience her gaze as something present. It seems that for some patients, the possibility of observing oneself on the screen evokes the experience of being looked at regardless of whether someone is actually looking at them. The other, whose look the patient experiences during teleconsultation, can be anonymous. As one participant (Thomas) says, “I have the awkward feeling that I am being constantly observed… I have the feeling that there are eyes everywhere and they are all looking at me.”

It should be pointed out, however, that the possibility of observing oneself does not always lead to self-objectification. As one participant (Dana) says:

I did not concentrate on the technology, on the fact that I am in a computer and that I can observe myself, instead I concentrated on the person and our conversation, on the object of the discussion.

Additionally, a correlation can be observed between one’s self-perception (whether it is positive or negative) and the tendency to observe oneself on the screen. If the patient has a negative self-image, then she tends to observe herself on the screen much more, shifting the focus away from the discussed problem to herself.^19^ One participant (Vilma) summarizes her experience by saying: “I can see myself [during teleconsultation] and I don’t know if I am ready to look at myself all the time in case I don’t like something.” Another participant (Thomas) illustrates his experience: “Overall – I don’t like how I look, I don’t like my voice, I don’t like anything about myself. The less I see of myself, the easier it is for me.”

Two additional things should be mentioned here. First, the possibility of self-observation is a feature of technology, which is usually easily avoidable; namely, most videoconferencing platforms offer the possibility of turning off or reducing one’s self-image. Second, apart from the already mentioned fact that self-observation in teleconsultation does not necessarily lead to self-objectification, it can actually be useful in some forms of clinical encounter (such as in some forms of psychotherapy, for example) as a part of the healing process. Taking this into account, I suggest viewing the possibility of self-observation as being neutral in itself, as it is a feature of videoconferencing technology that can be both positive and negative (or ‘good’ and ‘bad’), depending on the context.

## Conclusion: Transformation of the patient-health care provider relationship

Considering the fact that a major factor that influences the quality of patient‒physician relationships is the focus of the health care provider on the patient as a mere physical body that needs to be fixed, which leads to the objectification of the patient as a disease entity and the accompanying negative impact on the quality of care, in this paper, I focus on the question of what exactly happens to patient objectification in teleconsultation. Based on the results of my study of the patient experience of teleconsultation and on the work done within the phenomenology of medicine regarding the various sources of objectification, I attempted to determine if the patient experiences objectification during teleconsultation and, if so, what the source of this objectification is.

Referring to the two main sources of patient objectification in a clinical encounter, namely, the internalized gaze of the other and the medical technology itself, I argued that the more frequently mentioned main source of this objectification within clinical encounter, namely, the internalized gaze of the health care provider, is diminished in teleconsultation. This is the case for two reasons. First, due to the lack of a medical environment during teleconsultation, the patient perceives the health care provider as less intimidating, allowing the patient to be more open about her problems and to form a closer relationship with the health care professional, and leading to an increased sense of patient agency. Second, due to the absence of the patient’s body during teleconsultation, the health care provider has no other option than to focus on the story of the patient. Situating this shift of focus from the patient’s physical body to her lived body within discussions about the disease and the illness, I claim that in teleconsultation, rather than simply focusing on the disease of the patient, the health care professional focuses on the patient’s illness, thus avoiding treating the patient as an object and thereby cultivating an empathetic attitude toward the patient. Thus, paradoxically, the absence of the physical body in teleconsultation can advance the healing of the patient.^20^

In terms of the second source of objectification, namely, medical technology, I show that, without denying the fact that medical technology (such as X-ray images, for example) can be a source of objectification, there is another source of objectification present in teleconsultation, which is the medium of the videoconferencing platform itself. This is the case because it provides the possibility of the patient observing herself during consultation. I also argue, however, that the possibility of self-observation does not necessarily lead to the experience of objectification, and that it is easily avoidable, or, that most videoconferencing platforms offer the possibility of turning off or minimizing one’s self-image. Moreover, this self-observation experience can be used as a part of the healing process in some forms of clinical encounters. Taking this into account, I suggest considering the possibility of self-observation as being neutral in itself, becoming either positive or negative depending on context.^21^

While some patients experience objectification due to self-observation, I have argued that based on the results of the research study, it is the patient’s lived body and not their object body that is at the center of teleconsultation for most patients. This is evident in patients’ reportedly increased sense of agency during teleconsultation, and primarily, in their increased sense of control and responsibility. This increased sense of patient agency, together with a transformed attitude on the part of the health care professional (focusing on the patient’s lived body rather than object body) in the teleconsultation leads to both a diminished sense of the patient alienation and increased sense of closeness between the patient and the health care professional. This in its turn has an impact on the patient-health care professional relationship. The traditional hierarchical patient-health care provider relationship is transformed into a more horizontal relationship, where the patient plays a more active role. As one participant (Vilma) observes, “When I am with the doctor [in real life], I am under her rules and I have to follow them. The video format in some ways allows me to even these relationship out.” García et al. observed this transformation in the structure of an online clinical relationship in their study on online psychotherapy, describing it as “a more symmetrical interaction where the separation between roles becomes less sharp” (2022, p. 12). This disruption of the traditional hierarchical patient-health care provider relationship transforms the role of the patient from that of a passive recipient to that of a more engaged participant in her own healing process, thus increasing personal participation in the treatment process.^22^ Overall, the results of this study put into question the dominant view within the phenomenology of medicine, according to which the clinical encounter is viewed as a source of objectification for the patient. These results also support research arguing that the clinical encounter undergoes a significant transformation when it moves online. In addition, they challenge the claim that the lack of physical bodies in online clinical encounters leads to a disruption of the clinical relationship (Bizzari 2022). Without denying the fact that this shift online can have negative consequences for the clinical relationship due to the lack of physical bodies, the results presented in this paper show that this shift also has significant positive consequences, such as the diminished sense of alienation on the part of the patient and the increased closeness between the patient and the health care provider that occur in online clinical encounters.

This is not to say that all face-to-face clinical encounters should be substituted with online encounters, whenever possible. There are serious reasons (apart from medical necessity) against taking this approach. For example, people without technological skills and access to technology, as well as those who cannot express themselves verbally, would be excluded from receiving health care. In addition, the lack of physical bodies with the accompanying disruption of embodied features in teleconsultation (limited gestures and difficulty registering silences) can diminish a patient’s embodied trust in the doctor’s ability to heal (Bizzari 2022). Despite this, I think that online encounters illuminate some of the shortcomings of traditional on-site clinical encounters, such as the often encountered practice of treating the patient only as a disease entity and the strong emphasis on the passive role of the patient in her own healing process, and offer some insights for health care professionals into how to avoid or at least diminish these shortcomings, for example, by learning to listen to the patient’s illness story in addition to focusing on her diseased body, all of which can contribute to a more patient-centered level of care, both online and in person.

Finally, this study faces several limitations that should be taken into account. First, the generalizability of this study is limited for reasons pertaining to sample coverage and volunteer bias. Regarding the sample coverage, young adults and people with good-to-excellent digital skills were overrepresented. Older people and people without access to internet and computers did not participate in the study. With regard to the limited sample, it would be important to determine how people with low levels of digital proficiency experience teleconsultation, focusing on the question of the extent to which patients’ (as well as physicians’) digital proficiency influences online patient‒physician relationships. I suspect that the lack of digital proficiency (particularly on the part of the patient herself) would disrupt the patient’s experience of her lived body, thus leading to self-objectification. Further research is necessary to test this hypothesis.

Another limitation regarding the sample coverage lies in the fact that men and non-binary people were underrepresented in this study (among 14 respondents, only 3 were men, and none of the participants identified as non-binary). This limitation prevented the inclusion of a gender dimension in the analysis of the results. Future research by reference to more men and non-binary people would be interesting, as such research would allow us to examine patient objectification through the lens of gender differences. Furthermore, the generalizability of this study is limited because this study is specific to the health care system in Latvia. I focused on clinical relationships in the context of a health care system that is oriented on specific cultural values that are dominant in modern Western medicine. The whole question of patient objectification and its negative impact on the quality of care might not be relevant outside of the sphere of the influence of modern Western medicine.

The results presented in this paper also did not differentiate the patient experience of objectification based on the specialist that the patient visits. As the results of the study highlight the differences in patients’ experiences of teleconsultation associated with the specialty of the doctor with whom the patient is consulting (for example, regarding the importance of self-image), further research is needed to determine whether these differences are relevant to online patient objectification. Finally, while the focus of this research study was on patient experiences with teleconsultation, an increasing number of telemedicine tools are used in patient care, such as systems for remote patient monitoring. In this context, the question of patient objectification due to medical technology becomes particularly important and represents a significant topic for future research.

Lastly, the fact that participants of the study experienced teleconsultations during the COVID-19 pandemic, when they may have been reluctant to attend in-person appointments, may have influenced their experience. The fact that during the COVID-19 pandemic, the alternative to teleconsultation was not a face-to-face encounter but rather a mask-to-mask encounter should be taken into account, since in-person encounters were also modified in this context. Further studies are necessary to investigate whether the results regarding the experience of patient objectification in teleconsultation presented in this paper are transferable beyond the circumstances of the pandemic.

## Footnotes

1 Telemedicine literally means “healing at a distance” and, according to the definition provided by the European Commission (EC), is “the provision of healthcare services where traditional face-to-face patient‒doctor interaction (or doctor‒doctor) is replaced by over-distance interaction through the use of ICT.” (European Commission 2019).

2 The phenomenology of medicine is a heterogeneous field of research that uses concepts and distinctions drawn from the classical phenomenological tradition (e.g., embodiment, lived-body/object body, life-world, intentionality, body-schema, body-image, alienation) to gain insights into the patient’s lived experience of a variety of illnesses, disabilities and clinical encounters with the overarching goal of fostering the improvement of patient treatment and care (a few prominent phenomenologists working in this field are Richard Zaner, Drew Leder, Kay Toombs, Havi Carel, Fredrik Svenaeus, Luna Dolezal and Jenny Slatman).

3 While phenomenologists of medicine have not yet focused on the patient objectification that occurs in the context of *online* clinical encounter, some such researchers (for example, Luna Dolezal and Havi Carel) have expressed their views on the nature of online communication in general, arguing that it compares unfavorably to real-life, face-to-face interaction due to the former’s lack of the immediate presence of the body of the other (Carel 2020; Dolezal 2020). According to Dolezal, for example, in online interactions, we can never achieve the same levels of intimacy, closeness and contact that we can achieve in face-to-face interactions (2020, p. 23). While these thinkers have not addressed the issue of online patient objectification, the results presented in this paper put into question some of the insights expressed by Dolezal and Carel regarding the nature of online communication. More concretely, I show that at least online *clinical* encounters offer the possibility of establishing an even closer connection between the patient and the doctor than in-person, face-to-face clinical encounters.

4 While it is beyond the scope of this paper to provide an in-depth analysis of the differences in patient objectification between online mental health care and online physical health care, if any such differences are encountered in my research, I will highlight them.

5 For example, in multiple studies ranging across every medical specialty, researchers have observed that patients exhibit a high level of satisfaction with teleconsultations (Pogorzelska and Chlabicz 2022). Without denying the fact that there are various reasons for this high level of patient satisfaction with teleconsultations, based on the results of this study, I argue that the lack of patient objectification and the accompanying sense of closeness to the doctor and increased sense of the patient control definitely contribute to the positive experience that patients have with teleconsultations.

6 Focus on the patient as a mere object on the part of the health care provider is said to dehumanize the patient (Svenaeus 2021) and is mentioned by Marcum (2004) as a significant contributing factor to the quality of care crisis.

7 Drew Leder has pointed out (1990) that it is common in the phenomenological literature to overemphasize or even ontologize the distinction between *Leib* and *Körper.* I agree with Leder that the lived body and the physical body should not be interpreted as two different bodies: “*Körper* is itself an aspect of *Leib*, one manner in which the lived body shows itself” (Leder 1990, p. 6). In the context of this paper, however, I will reserve the concept of the lived body to refer to the experience of the body-as-it-is-lived-through, something which experientially coincides with the self and is absent in one’s everyday life. In contrast, the concept of the object body will refer to the *experience* of one’s body as an object, which presupposes an experiential distance between the body and the self and an explicit focus on the body.

8 The concept of alienation here refers to the bodily alienation understood as an experience of one’s own body as something alien to one’s self.

9 Svenaeus (2021) points out that objectification is a real problem in medicine and it can lead to bad medical practice or, in the worst case, dehumanization of the patient. However, he also emphasizes that not all objectifications are “bad” ones, leading to the experience of oneself as a mere object, some of them are “good” ones, retaining patient’s sense of subjectivity.

10 * See Table 1 for an overview of participants, including age, sex, length and number of teleconsultations, the specialist with whom they consulted, whether they had previous in-person familiarity with that specialist and the videoconferencing platform used.

11 While there are some differences in the experience of teleconsultation based on the type of the specialist visited (for example, a mental health versus physical health specialist or a primary care physician versus a specialist), these differences do not concern the transformation of the nature of the clinical counter online. Some of the differences have to do with a perceived lack of small talk, which is said to be a negative aspect in online psychotherapy and primary care (but a positive one when visiting a specialist), while others have to do with the importance of one’s body image in online psychotherapy rather than in cases of physical health consultations. In addition, patients experience an increased risk of being misunderstood in cases of consulting with physical health providers (versus mental health ones).

12 The use of online interviews has both benefits and limitations. It allowed flexibility in time and location, conformed to health and safety restrictions during COVID-19, and made it possible to observe participants using recorded video material. The main limitation of video-based online interviewing was restricting participation to those with access to the internet and a working computer, with good digital competencies, and with private space from which to participate in the interview. Other concerns include the security of the platform and patient confidentiality (for respondents at home or other environments where they can be overheard) (Lobe et al. 2020, p. 2).

13 Recently, Høffding and Martiny (together with Roepstorff) (2022) have addressed some objections to phenomenological interview as a source of reliable and valid knowledge.

14 For a more detailed account of how to conduct a phenomenological interview by integrating the qualitative interview with phenomenological philosophy, see Høffding and Martiny (2016).

15 As most participants of the research study were quite proficient in using internet and videoconferencing platforms, they did not experience loss of control in cases of technological disruptions. This does not mean however that it is not possible to have such an experience. Lack of technical knowledge and proficiency can definitely contribute to the feeling of a loss of control.

16 Cross-legged sitting position.

17 Two things should be mentioned here: first, this shift in attitude of health-care professionals is less significant in the case of psychotherapy, because the focus of in-person psychotherapy (at least in those forms that do not try to reduce mind to the workings of brain), to some extent at least, is already on the story of the patient (in addition to the nonverbal behavior of the person); second, while I want to maintain that a focus on the patient’s illness story is a welcome thing, it does not mean that there are no negative side effects to the absence of the patient’s physical body in teleconsultation. The lack of the patient’s physical body can disrupt the embodied features in teleconsultation (such as the limited gestures and difficulty registering silences), which in its turn can diminish patient’s embodied trust in the doctor’s ability to heal (Bizzari 2022).

18 We usually don’t have the possibility of looking in a mirror while we are talking to a doctor, and even if we did have this possibility, most people would not use it.

19 The study results indicate that self-observation is much more prominent within psychotherapy. This is also supported by observations made by Bizzari (2022) concerning autistic patents taking part in psychotherapy. Participants consulting with primary care physician or other specialists did not tend to observe themselves on the screen, instead focusing on the discussed problem.

20 Of course, this does not apply to cases where a focus on the patient’s physical body is of critical medical importance.

21 The conclusions in this paper regarding sources of objectification in teleconsultation can contribute to discussions about the alienating and objectifying nature of technology (Waldenfels 2011). While I have showed that technology can be a source of objectification (self-objectification), it (in the form of a video medium) can also diminish the experience of objectification for the patient, precisely because it facilitates a change in attitude of the health care professional toward the patient.

22 Despite the shift toward a more horizontal relationship, the health care professional still is in a position of power. This is because, although she cannot perform any physical manipulations during teleconsultation, she still retains the definitional power, meaning, that she still has the power to define the patient through diagnosis.
